# Monitoring of Food Waste Anaerobic Digestion Performance: Conventional Co-Substrates vs. Unmarketable Biochar Additions

**DOI:** 10.3390/foods10102353

**Published:** 2021-10-03

**Authors:** Nour El Houda Chaher, Abdallah Nassour, Moktar Hamdi, Michael Nelles

**Affiliations:** 1 Department of Chemical and Process Engineering, National Engineering School of Gabes, University of Gabes, Gabes 6029, Tunisia; 2 Department of Waste and Resource Management, Faculty of Agricultural and Environmental Sciences, University of Rostock, 18051 Rostock, Germany; Abdallah.nassour@uni-rostock.de (A.N.); Michael.Nelles@uni-rostock.de (M.N.); 3 Department of Biological and Chemical Engineering, National Institute of Applied Sciences and Technology, University of Carthage, Tunis 1080, Tunisia; moktarhamdi11@gmail.com; 4 DBFZ German Biomass Research Center GmbH, 04347 Leipzig, Germany

**Keywords:** food waste treatment, co-substrates, anaerobic co-digestion, organic loading rate, AD-effluent quality

## Abstract

This study proposed the selection of cost-effective additives generated from different activity sectors to enhance and stabilize the start-up, as well as the transitional phases, of semi-continuous food waste (FW) anaerobic digestion. The results showed that combining agricultural waste mixtures including wheat straw (WS) and cattle manure (CM) boosted the process performance and generated up to 95% higher methane yield compared to the control reactors (mono-digested FW) under an organic loading rate (OLR) range of 2 to 3 kg VS/m^3^·d. Whereas R3 amended with unmarketable biochar (UBc), to around 10% of the initial fresh mass inserted, showed a significant process enhancement during the transitional phase, and more particularly at an OLR of 4 kg VS/m^3^·d, it was revealed that under these experimental conditions, FW reactors including UBc showed an increase of 144% in terms of specific biogas yield (SBY) compared to FW reactors fed with agricultural residue. Hence, both agricultural and industrial waste were efficacious when it came to boosting either FW anaerobic performance or AD effluent quality. Although each co-substrate performed under specific experimental conditions, this feature provides decision makers with diverse alternatives to implement a sustainable organic waste management system, conveying sufficient technical details to draw up appropriate designs for the recovery of various types of organic residue.

## 1. Introduction

In recent times, concerns over the status of conventional sources of energy such as fossil fuels have steered the world in the direction of unconventional sources. In fact, the limited resources for the generation of traditional forms of energy have not only underlined the need for sustainable alternatives, but have also drawn attention to the alarming impact of coal, oil, and natural gas usage on global climate change [1]. Hence, it makes sense in the modern world to switch to profitable and sustainable substitutes [2]. To this end, different options have been evaluated in order to identify those most appropriate as sustainable sources of renewable energy. Wind, solar, and biomass have been classified as fundamental sources of non-fossil-oriented energy, and were envisaged as becoming the most prominent sources of renewable energy in the world [3]. In this framework, particular attention was paid to the conversion of biological materials into bioenergy, outlining their valuable contribution to offsetting the shortfall created by the reduction in the amount of fossil energy. Moreover, biological treatment seems to be an alternative worth considering in that, in addition to the efficient recovery of generated waste, there is the possibility of economic profit, as well as the sustainability of existing waste management systems [4]. As the exploited biomass might be transformed into energy with several technologies, bioethanol, biodiesel, biogas, and even biochar are envisioned by-products that can be tapped eventually as powerful green energy resources [5].

Biogas, as one of the above-mentioned by-products, is the principal product gathered as a result of anaerobic digestion (AD) and can either be utilized forthwith or converted into other forms of energy [6,7]. Supplying clean energy is not a unique feat of anaerobic treatment. The latter has been given high priority over the past 10 years as it has been identified as an eco-friendly option that can be widely utilized to reduce organic waste volume, which in turn leads to a decrease in greenhouse gas emissions [2,8]. However, both nitrogen-rich materials such as food waste or manure, and carbon-rich materials such as lignocellulosic residues, are suitable for feeding anaerobic reactors [9]. To this end, substrates availability is considered one of the key factors when selecting the appropriate organic waste to be treated. Thus, due to its high energy content and wide availability, food waste (FW) has attracted worldwide attention as a material for anaerobic processing [10,11]. Nevertheless, adopting AD for FW management still encounters numerous technical and economic limitations, as FW is generally constituted of easily degradable components entailing process acidification and a weak buffer capacity, causing process inhibition [12]. In order to overcome these concerns, approaches such as reducing the organic loading rate (OLR) [13], pH regulation [14], and co-substrate addition, are usually adopted [15]. Several works have identified the challenges associated with FW anaerobic treatment and have revealed that anaerobic co-digestion (ACoD) is one of the most appropriate alternatives, particularly when the selected co-substrates are adaptable in order to compensate for FW mono-digestion deficiency by improving the buffer capacity and balancing the beneficial nutrients [7,16].

Recognized as one of the most common classic co-substrates used during anaerobic treatment, agricultural wastes are categorized as a relevant and beneficial booster of FW-AD. Moreover, the agricultural sector generates various types of residue, ranging from highly nitrogenous types such as animal feces to the poorest types such as straw, which makes the sector an opportune supplier of biowaste for anaerobic treatment [17]. Focusing on nature on the one hand, and the abundance of organic residues generated from agricultural activities across the world on the other, cattle manure (CM) has been identified as one of the most extensively disposed substrates that could be biologically recovered [14]. Several researchers have opted to exploit CM as an effective co-substrate to improve FW-AD, pointing out the high synergy between FW and CM, which subsequently ensures efficient biodegradation once the feedstock mixture is used under suitable operational conditions [16,18]. In the same context, Zhang et al., (2013) reported that the increased buffer capacity supplied by CM allowed the technology to proceed at relatively high organic loads, which was generally unfeasible for mono-digested FW, making the addition of manure a convenient booster for the anaerobic treatment of FW [19]. However, despite all the above-referenced benefits, CM is characterized by a relatively low carbon to nitrogen ratio (C:N), which limits, to a certain extent, its effectiveness as a co-substrate for AD, particularly when FW is also qualified by the same C:N ratio range [20]. Therefore, a supplement in the form of another organic substrate is indispensable. To this end, a second agricultural residue, characterized by possessing a significant ratio of carbon, might be exploited to meet the required criteria [21]. In this regard, different studies have indicated that straw is identified as one of the optimal alternatives to improve the initial C:N ratio of FW, and to then guarantee the steadiness of the process. Furthermore, Kaldis et al., (2020) reported that wheat straw (WS), despite having a rigid structure that requires a specific pretreatment, is considered as a favorable substrate for biogas production [22]. In addition, Zahan et al., (2018) evaluated the effect of substrate mixtures including FW, WS, and chicken manure on AD performance, and revealed that the adjustment of the initial C:N ratio was crucial to ensure a longer performant process with higher methane and biogas production as well as highly qualified digestate [23]. Hence, the agricultural sector seems to be as an excellent conventional supplier of efficient FW-AD co-substrates as it furnishes the reactors with the required features: high buffering capacity and an equilibrated C:N ratio [24,25].

Nevertheless, within the scope of both a sustainable waste management system and effective FW anaerobic treatment, further sectors of activity might also present a potential source of appropriate organic waste for the ACoD of FW. Indeed, as the reactors require a carbon-rich additive capable of balancing the initial C:N ratio, as well as a buffering regulator, unmarketable biochar (UBc), which went from a valuable by-product to industrial waste, might possess the required characteristics [26]. In this context, several research works pointed out that the amendment of anaerobic reactors with carbonaceous materials, including biochar and activated carbon (AC), might be effective due to their positive impact on biogas generation and their widespread accessibility [27,28,29]. To this end, Rasapoor et al., (2020) investigated the effects of different AC and biochar additions on biogas generation yield during AD of complex organic waste rather than mono-substrate waste and revealed that biochar improved the methane yield and adsorbed ammonia nitrogen better than AC [30]. In fact, this might be due to the potential of biochar to enhance the biodegradation performance, as it is characterized by a porous structure favoring the colonization of functional microbial communities [29,31]. From a biological point of view, biochar was considered as having a microbial-support function by exchanging electrons from a donor to an acceptor [32]. Consequently, biochar was touted as a good conductive material for stimulating direct interspecies electron transfer (DIET) between methanogens and exoelectrogenic microorganisms coexisting in anaerobic reactors [33]. Additionally, biochar with its porous structure was deemed to offer great support in terms of boosting methanogen development during the AD process [34]. When it comes to the technical side, diverse researchers have confirmed that carbonaceous material has been widely tested to reinforce buffering capacity, delay the accumulation of volatile fatty acids (VFAs), and mitigate ammonia inhibition, which strengthens the durability of the AD process [35]. In the same context, Zhang et al., (2020) linked the biological and physiochemical aspects, and reported that biochar’s high surface area ensured a high degree of inhibitive compound adsorption, reduced acidification, and then ensured a high-performing process, as well as highly qualified AD effluents [36]. Admittedly, while biochar addition has significant benefits in terms of enhancing anaerobic treatment, it is also worth mentioning that the nature of the initial feedstocks used, as well as the selected process and the operational conditions, ultimately raise further concerns in relation to UBc exploitation. Indeed, despite the fact that UBc has almost the same characteristics as the biochar produced (e.g., in terms of density, surface area, and pore size), UBc-residue upcycling is still relatively limited. Few researchers have tested the effect of biochar size on AD performance. However, Zhang et al., (2020) revealed that biochar with different particle sizes (<50 μm to 3 cm) substantially improved methane yield [36]. Accordingly, UBc-residue upcycling as a co-substrate of FW anaerobic treatment was tested during the experimental work described in this paper to identify whether or not the unmarketable carbonaceous material could provide the digester with the required features.

The main objective of this research study was to improve the anaerobic treatment of FW, exploiting various organic residues gathered from different activity sectors in agriculture and industry. To achieve this, the rationale was to balance the initial C:N ratio, as well as the macro and micro-nutrients, to evaluate the performance of anaerobic co-digestion under a stable organic load as a first step, and then under variable OLRs. Therefore, the outcomes of this research can be exploited as guidance, highlighting the potential efficiency of FW anaerobic co-digestion intended for implementation in different areas. In addition, it sought to provide sufficient technical detail to design such facilities.

## 2. Overall Concept

**Figure 1:**
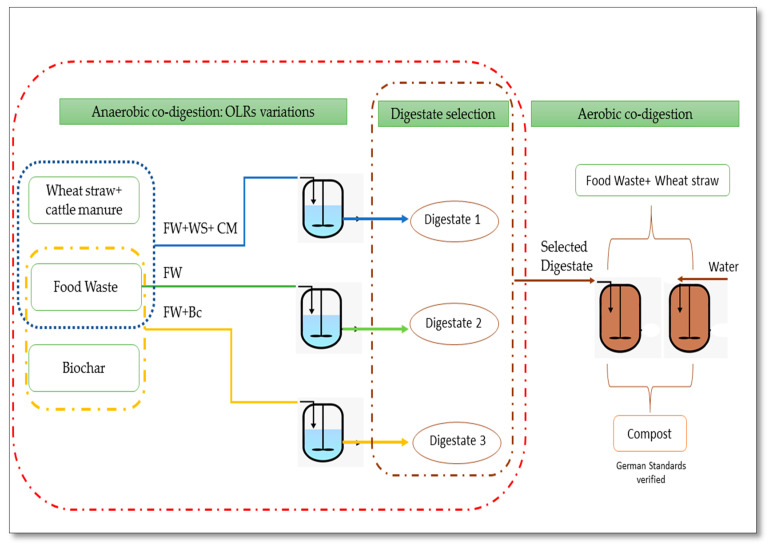
Conceptualization of the overall Renew_Value approach.

The research work was launched using the Renew Value project framework, aiming to boost the recovery of FW combined with other forms of organic residue generated from different activity sectors. The overall concept followed in the project is illustrated in Figure 1. During the experimental work, FW was subjected to consecutive biological treatments: aerobic and anaerobic digestion. Therefore, the work was fundamentally divided into two phases. During the first phase, the main input material used, FW, was separately mixed with various co-substrates generated from different sectors—industrial and agricultural. Wheat straw (WS) and cattle manure (CM) were selected to be part of the agricultural residue. Indeed, WS is known to be a carbon-rich material, while CM is rich in nitrogen, which gives their combination pairing the required AD features. When it comes to the industrial area, biochar was chosen to be evaluated as a FW co-substrate feeding anaerobic digesters.

In this approach, the biowaste for use was selected on the basis of the potential of the selected co-substrates to enhance the starting parameters of the FW anaerobic treatment, as well as the process efficiency. Hence, a comparison between the agricultural residue (CM and WS) addition effects, as conventional co-substrates to FW anaerobic co-digestion, as well as the biochar impact as an unconventional type, was performed.

It is worth mentioning that over the anaerobic process, pH was maintained without any adjustment to evaluate its effect on the progress of AD operating under variable OLRs, as this might be the most appropriate way for a rough estimation of real conditions met on a large scale.

## 3. Materials and Methods

### 3.1. Substrate Selection

**Figure 2:**
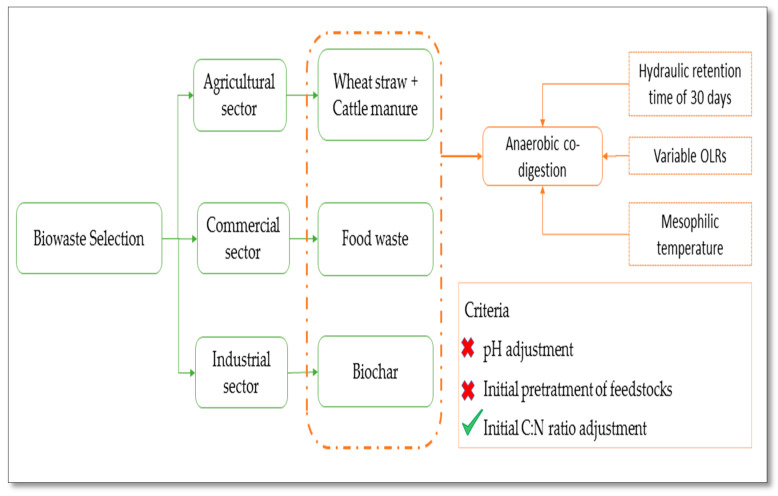
Project requirements and approach.

The main target of the experimental work was to enhance the biological treatment of organic residue. As FW is abundantly generated from different sectors including the hospitality industry, the agri-food sector, the commercial sector, etc., FW was chosen as the principal component [24]. However, certain criteria were considered to ensure a rigorous selection of the co-substrates to be used (Figure 2):

- Exploitation of different types of organic residues gathered from different sectors of activity: WS and CM as two of the most abundant residues generated from the agricultural sector [24], and biochar as a recoverable solid residue produced by industry [29];
- Potential of the added organic materials (UBc or WS plus CM) to enhance the initial C:N ratio, process performance, as well as the AD-effluent characteristics: biogas and digestate in line with previous work [16,37].

Accordingly, four types of organic waste were selected as feedstock materials for anaerobic reactors; FW was used as the main substrate, while CM and WS were mixed to be exploited as potential co-substrates, with UBc utilized as a valuable additive, boosting a semi-continuous anaerobic co-digestion process.

### 3.2. Samples and Inoculum Preparation

Food waste was mainly composed of rice, noodles, salads, and bread, and was initially collected at a university canteen and then stored at −20 °C to stop any biological reaction, while wheat straw was stored in plastic airtight buckets at ambient temperature. To ensure a good mix, the volumes of the WS and FW were reduced using a lab blender (GRINDOMIX, Retsch GmbH, Germany), while cattle manure was kept in its raw state. Biochar was obtained from an industrial manufacturer of charcoal, made using natural beechwood. Biochar consisted of undersized particles (<5 mm) that were not marketable.

The start-up of an anaerobic digester is significantly influenced by the quality of the inoculum used, as it plays a crucial role in supplying the reactors with acclimatized microorganisms, as well as the required trace elements [38]. Therefore, to establish the desired anaerobic start-up conditions, the inoculum was collected from a biogas plant treating FW under mesophilic conditions. At the beginning of the process, the inoculum was maintained anaerobically at 37 °C for five weeks to minimize background biogas production [39].

### 3.3. Experimental Setup

The experiments were carried out in mesophilic lab digesters with a nominal volume of 20 L. The digesters were heated by warm air, and kept at a constant temperature of 38 ± 1 °C. An internal stirrer (anchor type) was installed in each digester. Each mixture was stirred for 5 min every 30 min at an approximate speed of 80 rpm. Reactors were equipped with inlet and outlet valves for feeding and for digestate withdrawal. Except on weekends, the reactors received different mixtures of organic waste, twice per day. The hydraulic retention time (HRT) was maintained for 15 days [40,41]. The experiment lasted for almost 7 months, and was divided into two phases. Phase I was devoted to the acclimatization of microorganisms and the start-up of reactors. During this phase, the organic loading rate (OLR) was kept stable at 2 kg VS/m^3^·d. Phase II was characterized by a progressive increase in OLR with a stepwise increment of 0.5, ranging between 2.5 and 4.5 kg VS/m^3^·d (Table 1).

Each tested set of parameters was applied in duplicate, comprising:

- R1: 100% FW;
- R2: 60% FW+ 20% CM+ 20% WS (*w*/*w*);
- R3: 90% FW+ 10% UBc (*w*/*w*);

Digester feedings as well as AD performance monitoring, including the determination of biogas composition VFAs, TAC, pH, etc., were stopped if one of the following criteria indicating an unsuitable environment for anaerobes development existed:

- % CH4 lower than 50% [16];
- VFAs: TAC > 0.4 [42];
- pH < 6.5 [40];

To assess process performance, samples of digester content were taken once per week via the feeding port to identify different physiochemical properties such as moisture content (MC), total solids (TS), volatile solids (VS), volatile fatty acids (VFAs), alkalinity (TAC), and pH. In addition, the biogas volume was monitored online using drum-type gas meters (type TG05, RITTER Mess Technik GmbH, Germany) while gas volume was logged continuously. To analyze the biogas composition, the headspace of each digester was analyzed every other day using a gas analyzer type EHEIM VISIT 30 (Eheim Mess Technik GmbH, Germany). Consequently, CH_4_, CO_2_, H_2_S, and O_2_ components were identified.

### 3.4. Analytical Methods

FW, WS, CM, UBc, and the collected digestates D (i = 1,2,3) were characterized by measuring different physical-chemical parameters. MC, TS, and VS contents were determined gravimetrically, following CENT/TS 14744-1 (2009). In addition, the C:N ratio and major and minor mineral content analyses of all the substrates were determined in an external laboratory following the methods described in EN ISO 16967 (2015) and 15297 (2011), respectively (Table 2).

Regarding operational parameters for assessment of anaerobic digestion stability, VFAs, alkalinity, and pH were determined using an automated titration unit (Titra-Lab 1000, Hach Instruments Germany), which involved centrifuging a digestate sample at 4000 rpm for 30 min to obtain a supernatant. Then, 5 mL of the supernatant was used for a titration with 0.1 mol/L sulfuric acid until that pH value attained 5, in accordance with USEPA (1983). The volume of biogas was normalized to standard conditions comprising dry gas, standard temperature, and pressure (0 °C and 1 bar) according to the method described by Somashekar et al., (2014), the results of which are presented as normal-liters (L_N_) [43].

### 3.5. Statistical Analysis

Statistical analysis was conducted using XLSTAT 2021 with one-way analysis of variance (ANOVA) and the Tukey method with a probability level of 0.05 to evaluate the effects of co-substrate addition on specific biogas yield (SBY) during Phase I, while the effects of multi-factors and their interactions on dependent variables during Phase II were conducted using multivariate analysis of variance (MANOVA). Therefore, Wilks tests were used to identify λ and *p*-values.

## 4. Results and Discussion

### 4.1. Properties of the Raw Material

The physical and chemical characteristics of the residues are summarized in Table 3. The moisture content was found to be approximately 74.0%, 2.4%, 92.9%, and 8.9% of the fresh matter, leaving behind dry matter content of 26.0%, 97.6%, 7.1%, and 91.1% for FW, UBc, CM, and WS, respectively. Because microorganisms as well as anaerobic digestion systems have a certain demand for carbon and nitrogen in any growth environment, the C:N ratios were evaluated for each substrate with the exception of the biochar. Because the nitrogen content of UBc was undetectable, its initial C:N ratio could not be determined. However, the C:N ratios of FW, CM, and WS were initially measured at 17.10, 25.64, and 78.08, respectively. In addition, the minor minerals in the form of micronutrients or essential supplements were examined, as they are crucial for the functioning of the methanogenic bacteria [12]. Therefore, certain minor elements such as Cu, Ca, Pb, and Zn, as well as some major constituents such as P, K, and Mg, were monitored. The characteristics of the various substrates analyzed compare closely with the reported literature [45].

### 4.2. Effect of Co-Substrate Addition on Startup Conditions of FW-ACoD

To overcome the inhibition of the anaerobic process, the C:N ratio is considered as one of the key factors to be initially set at the required value [46,47]. To this end, from the beginning of the experimental work, FW, WS, CM, and UBc were analyzed to determine the carbon and nitrogen concentrations (Table 3). Indeed, the C:N ratio identified for different organic materials proved that the abundance of nitrogen content, particularly for FW and CM or the carbonaceous aspect of WS and UBc, made those residues unsuitable for anaerobic mono-digestion [48]. Therefore, different substrates were combined to regulate the C:N ratio of each feedstock mixture within the required range of 20–40 [20]. Digesters fed only with FW and R1 (i.e., FW_100_) were characterized by a C:N ratio of 17.10, while the C:N ratios of the co-digested substrates increased from 17.10 to 29.64 for R2 (FW_60_CM_20_WS_20_) and from 17.10 to 37.88 for R3 (FW_90_UBc_10_). Thereafter, the effect of the C:N ratio regulation by mixing different types of organic residue was evaluated based on the progress of the process during the start-up conditions.

**Figure 3:**
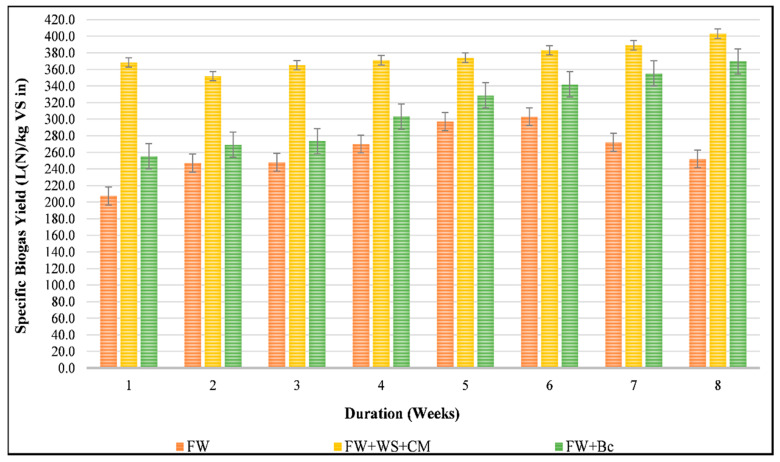
Effect of mixing substrates on specific biogas yield during the start-up conditions.

**Figure 4:**
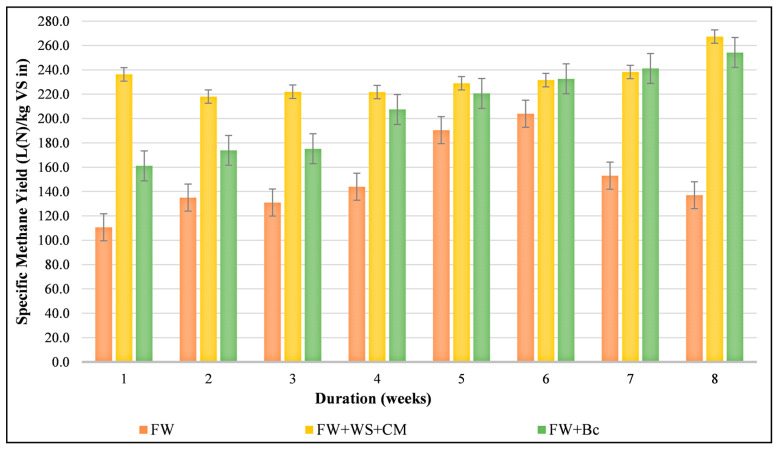
Effect of mixing substrates on specific methane yield during the start-up conditions.

Figure 3 illustrate the specific biogas yields generated from different feedstock mixtures (R1, R2 and R3) during Phase I of AD, which was characterized by a stable organic loading rate (OLR) of 2 kg VS/m^3^·d. Regarding the mono-digested FW, the biogas production as well as the measured methane rate were almost stable during the first 4 weeks at around 243.1 L_N_/kg VS_in_ and 55%, respectively. However, by the end of the fifth week, the biogas and methane yield improvements were considerable; SBY and SMY reached around 303.7 and 204.1 L_N_/kg VS_in_, respectively (Figure 4). It is worth mentioning that the highest methane production (of around 67.32%) was only noticed from the sixth week of running R1. In fact, the R1 performance was expected, and might be due to the lagged acclimatization and adaptation of the coexisting microbial community with the mono-digested FW because this latter was characterized by a relatively low C:N ratio and an acidic pH. In this regard, Xu et al., (2018) reported that FW properties significantly affected the adaptability of microorganisms to the availability of beneficial or inhibitor micro-and macro-nutrients during the anaerobic mono-digestion of FW, thereby impacting the process’s durability [10]. Moreover, by the end of the eighth week, R1 productivity dropped by 33% in terms of methane yield, while the inhibition indicators (VFAs, TAC, pH, etc.) did not show any kind of process failure, promoting the gradual increase in the OLR for R1.

For the amended reactors, Figure 3 shows that from the first week of the start of the experiment, a significant SBY improvement was recorded at around 77% for R2, while a lower biogas production enhancement was seen in the case of R3 (around 23%). Indeed, the recorded specific biogas yields, particularly during the first week, revealed the close nexus between the initial C:N ratio of the feedstocks and their productiveness. Therefore, the more suitable AD-C:N ratio that marked R2 (a C:N ratio of 29.64) led to more biogas production than in the case of R3 (with a C:N ratio of 37.88). These findings were in line with those of Choi et al., (2020), indicating that, for a significant ratio of C:N exceeding 35, acidogenic bacteria promptly consumed the available nitrogen, leading to a lower biogas yield as in the case of R3 [49]. However, in terms of biogas composition, R2 and R3 generated a comparable methane rate of about 63%. Hence, the combination of WS and CM definitely promoted the initial C:N ratio and even enhanced biogas production, but the addition of biochar significantly influenced the composition of the generated biogas, particularly the production of methane [33,50]. By the end of Phase I, both R2 and R3 witnessed higher biogas production, reaching 403 L_N_/kg VS_in_ and 369.7 L_N_/kg VS_in_, qualified by 66.33% and 68.77% of CH_4_, respectively. Therefore, as shown in Figure 4, the methane yield enhancement was around 86% for both R2 and R3 compared to R1. Moreover, during Phase I, the statistical analysis ascertained the significant effects of different co-substrates with SBY, either agricultural residue or commercial waste (*p*-value < 0.05), indicating the effective anaerobic co-digestion of FW.

Obviously, balancing C and N and ensuring sufficient buffering capacity are some of the factors that promote FW anaerobic co-digestion [42,51]. However, digesters treating only FW revealed insufficient trace metal content, which is considered an essential growth factor for anaerobes [52]. Therefore, instead of placing doses of the required micronutrients into the digester, relying on the existing trace elements content (TEs) in the feedstocks might be an option [53]. Accordingly, the stable anaerobic process, and the promoted methane production that marked R2 and R3 compared to R1, were due not only to the adjusted C:N ratio, but also to the potential of CM and UBc to compensate for the deficit in terms of macro and micro-nutrients [12]. Therefore, the supply of certain nutrients ensured by the addition of both CM and UBc enhanced the buffering capacity by providing a suitable concentration of calcium (Ca), of around 0.61 g/kg TS and 0.57 g/kg TS, respectively [15], boosted the growth of all the methanogens by supplying 6.91 g/kg TS and 9.34 g/kg TS of Nickel (Ni) [54], and strengthened the process performance by adjusting the different rates of further required TEs [55]. Hence, FW anaerobic co-digestion with different types of organic residue generated from different sectors of activity seems to be a good alternative to simultaneously upgrading sustainable organic waste management, as well as a performant anaerobic digestion process under stable OLR.

### 4.3. Effect of Co-Substrate Addition on ACoD Performance under Variable OLRs

#### 4.3.1. Early Warning Indicators for FW-ACoD Monitoring under Variable OLRs: Specific Biogas Yields vs. VFAs, Alkalinity, and VFAs: TAC Ratio

**Figure 5:**
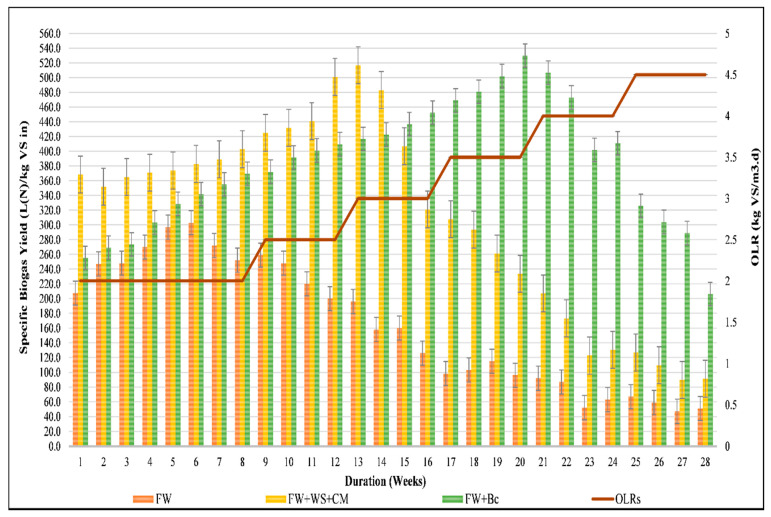
Specific biogas yields of different mixtures at different OLRs.

Since the HRT and OLR were considered as key factors in process stabilization, a balanced HRT and OLR were taken into consideration during Phase II, which was characterized by a variation of OLRs every 4 weeks [40]. As the fluctuation of OLRs ranged from 2.5 to 4.5 kg VS/m^3^·d with a pace of 0.5, these increments were divided into four categories to assess the impact of OLR variations on the process evolution. Figure 5 illustrates the specific biogas production recorded during Phase II with regard to each feedstock mixture. Starting with R1, the increase in OLR to 2.5 kg VS/m^3^·d was not initially significant in terms of SBY, as digester productivity was almost stable (±2%), particularly during the first 2 weeks of Phase II. However, by the end of the first feeding cycle (week 12), R1 was marked by a decline of 20.6% in SBY. Moreover, the measured volatile fatty acids (VFAs) and total alkalinity concentration (TAC) during Phase II revealed that the noted drop in biogas production was due to the VFAs and TAC behaviors (Table 4). The concentration of VFAs reached around 123% of the increase from the end of Phase I to the end of the first feeding cycle of Phase II (OLR of 2.5 kg VS/m^3^·d), while simultaneously a decrease in TAC of about 40% was also observed. Indeed, as FW was characterized by an abundance of easily degradable components, specifically during the sensitive microorganism acclimatization period in the reactor environment, a prompt accumulation of VFAs and a drop in TAC were anticipated. The current findings were in conformity with the findings of several researchers who reported that, in the case of FW mono-digestion, 2 to 2.5 kg VS/m^3^·d was deemed to be an optimal OLR range for improving overall system performance in terms of stability, productivity, and efficiency [12,13]. Furthermore, with a continuous rise of OLRs, an intense decline in SBY was logged, with a fall to 200 L_N_/kg VS_in_ and then to 126 L_N_/kg VS_in_ for 2.5 and 3 kg VS/m^3^·d, respectively, indicating the inhibition of R1. Table 4 shows that, from the first feeding cycle to the end of the second one, a significant VFAs accumulation of up to 194% coincided with a considerable decrease in TAC (of around 77%), which indicated R1 failure. Regarding the VFA:TAC ratio, this was around 0.17 ± 0.04 during Phase I, indicating that the OLR might be safely increased [42]. However, from the beginning of Phase II, the VFA:TAC ratio increased to 0.37 at an OLR of 2.5 kg VS/m^3^·d, and reached 1.89 ± 0.98 once the OLR rose to 3 kg VS/m^3^·d, confirming that R1 was overloaded, and mono-digested FW failed under an OLR higher than 2.5 kg VS/m^3^·d [13]. In the same context, Kumar et al., (2015) revealed that under an OLR higher than 2 kg VS/m^3^·d, an intensive concentration of propionate hardly convertible to acetate caused FW anaerobic digestion failure, which might explain the R1 behavior during the current research [56]. Therefore, focusing on the OLR limitations, particularly for a large scale, mono-digestion of FW was more resistant with moderately low and constant OLRs [57,58].

One of the main objectives of this experimental work was to evaluate the effects of agricultural and industrial waste on FW anaerobic process performance operated at different OLRs. Figure 5 shows that SBY profiles changed during the different experimental phases, as well as the nature of the feedstock used. At an OLR of 2.5 kg VS/m^3^·d, R2 demonstrated an improvement of 24% of SBY during the transitional period from Phase I to Phase II, while R3 showed only a 10% increase, a feature that could be due to the basic properties of the co-substrates used [59]. For that purpose, Zahan et al., (2018a) highlighted that an optimum carbon concentration, as well as the use of macro and micro-nutrients, had a positive effect on avoiding excessive ammonia inhibition during FW, WS, and chicken manure anaerobic treatment under variable OLRs [23]. UBc and WS are ultimately carbonaceous substrates, and with reference to Zahan et al., (2018a), this absolutely implies an unstable process for both R2 and R3, whereas the addition of CM boosted R2 progress during the start-up and the first weeks of the transitional phase, creating a difference in terms of biogas generation between R2 and R3. However, from week 15 onwards, at an OLR of 3 kg VS/m^3^·d, the SBY tendencies of R2 and R3 were slightly reversed (the λ-value was around 0.866). Furthermore, a difference of around 21% was shown in terms of methane volume gathered from R3 compared to R2. However, it is also worth mentioning that under those experimental conditions, R2 presented the highest SBY improvement compared to mono-digested FW, attaining 213% (the λ-value was around 0.514).

Although highly similar VFA tendencies were identified for R2 and R3 from the initial start-up phase until an OLR of 3.5 kg VS/m^3^·d, biochar addition influenced VFA alleviations. In fact, an almost stable VFA concentration was notable until the end of the second feeding cycle (OLR of 3.5 kg VS/m^3^·d), matching a maintained R3 alkalinity, which ranged from 6000 to 7500 mg CaCO_3_/L (Table 4). This latter gave rise to an enhanced SBY (the λ-value was around 0.367), reaching 152% and 126% marked R3 compared to R1 and R2, respectively. In addition, the alkalinity trends of R3 might be linked to some specific properties of biochar, including those of soluble ash, fixed carbon, or volatile matter content, and might significantly boost the stability of FW anaerobic reactors [60]. The current findings were in line with the results reported by Giwa et al., (2019), confirming that the alkaline nature of biochar considerably influenced the AD alkalinity and upgraded the in-situ biogas quality by reacting with CO_2_ and H_2_S [61].

Reaching the third feeding cycle of Phase II, R2 showed a decline in terms of SBY (around 42%) and TAC concentration (around 33%), whereas VFAs accumulated (around 39%) and impacted the VFA:TAC ratio tendencies. In fact, the continuous increase in the VFA: TAC ratio from 0.67 to 0.89 revealed that R2 was overloaded, marking the process failure of the digesters fed with mixed food waste and agricultural residue, at an OLR of 4 kg VS/m^3^·d. On the other hand, relatively stable performance of R3 was observed under the same experimental conditions with a VFAs concentration of 3115 mg/L, and a total alkalinity of about 5840 mg CaCO_3_/L, indicating that the system was not inhibited by acidification, even if the VFA: TAC ratio overstepped the upper bound of the overloading value by attaining 0.53 [62]. In the same context, Ma et al., (2020) reported that accumulated acids were readily converted to biogas in the presence of biochar as an additive because such a buffering agent might be efficient in terms of reducing VFA accumulation by boosting the direct interspecies electron transfer (DIET) functions between acidogenesis and methanogenesis microorganisms [63]. Although R3 illustrated resistant AD progress from the first to the third categories of Phase II, the fourth category (at an OLR of 4.5 kg VS/m^3^) outlined an increased VFA:TAC ratio of about 1.21, which was due to an increased VFAs concentration at about 4051.25 ± 276.52 mg/L, and decreased alkalinity of around 3351.00 ± 437.15 mg CaCO_3_/L. Simultaneously, a gradual drop in SBY, by approximately 50% from week 24 until the end of the running of the digesters, was recorded. Hence, VFA and TAC behaviors were also consistent with the trends in biogas production, where well-buffered digesters generated improved volumes of biogas [64,65]. In this sense, the operational conditions, particularly the feedstock mixture and the OLR variations (*p*-value < 0.0001), significantly influenced the efficiency of the existing microorganism community in terms of sustaining AD progress and converting polymers to methane.

#### 4.3.2. Early Warning Indicators for FW-ACoD Monitoring under Variable OLRs: Specific Methane Yields vs. pH Fluctuations

R2 and R3 showed improved biogas production during the entire period of anaerobic treatment. However, at different OLRs, mixed feedstocks that were marked by enhanced SBY did not inevitably produce better SMY. Table 5 shows that R3 produced the greatest rate of methane overall throughout the process while, from the beginning of the digesters’ run until the second feeding cycle of Phase II (OLR of 3 kg VS/m^3^·d), R2 provided higher biogas production (Figure 5). Nevertheless, it is also worth mentioning that, at a certain point, a considerable SMY was also generated from food and agricultural waste mixtures. Indeed, more methane was generated by R2 compared to R1, reaching around 95%, 229%, and 376% of amelioration at OLRs of 2, 2.5 and 3 kg VS/m^3^·d, respectively. This advancement was originally due to the balance of the C:N ratio ensured by both WS and CM. However, this might play a key role by providing the methanogens with some of the trace elements required to stimulate the activity of the enzymes and co-enzymes for better methane production, and particularly by buffering the digesters at certain organic loads [10,64]. With regard to these experimental conditions, R2 was characterized as having a stable pH and, consequently, constant VFA levels. Table 5 shows that a neutral pH range, coupled with almost stable VFAs and TAC, were logged for OLRs ranging from 2 to 3 kg VS/m^3^·d, and ensured a high methane content of around 65%. However, from the third feeding cycle of Phase II, a significant drop in SMY of approximately 46% marked R2, and impacted the biogas quality generated (CH_4_ of 55%). Though pH is not an early indicator of process steadiness, it is crucial to control also the VFAs to alkalinity ratio to decide about feeding the digester. From an OLR of 3.5 kg VS/m^3^·d, the VFA:TAC ratio was around 0.67 ± 0.04, associated with a fall in pH over a value of 6, thereby hindering methanogen growth, leading to an excessive amount of CO_2_ formation and causing R2 inhibition [31]. Similar findings were found by Hegde et Trabold, (2019) who reported that the pH of the substrate used supported a faster acclimatization of the microorganisms [65], allowing easy uptake of certain required nutrients, and revealed that the maximum methane yield was observed for mono-digested and co-digested FW, with CM under an OLR of 2.8 kg VS/m^3^·d, and for a range of pH from 6.8 and 7.3. In the case of R3, permanently increased SMYs were logged from Phase I to an OLR of 3.5 kg VS/m^3^·d. Under these operational conditions, pH fluctuated from 7.49 ± 0.39 to 7.91 ± 0.20, which was explained by the available biochar surface areas to be colonized by bacteria and methanogens, promoting VFAs consumption [50]. However, Zhai et al., (2020) revealed that rapid VFAs utilization characterized the amended reactors using biochar, which was not really in accordance with the current results, as the VFAs concentrations were almost stable until an OLR of 3.5 kg VS/m^3^·d was achieved [65]. Nevertheless, the alkalinity tendencies of R3 were in line with those of different studies testing biochar efficiency with regard to anaerobic treatment [36,58]. Table 4 illustrates that high alkalinity marked R3, which varied between 10207.44 ± 2531.58 and 5842.50 ± 398.62 mg CaCO_3_/L from Phase I to the third feeding cycle of Phase II (OLR of 3.5 kg VS/m^3^·d). Indeed, the notable alkalinity of R3 was definitely linked to the alkaline nature of the biochar, which promoted methane formation by causing CO_2_ and H_2_S to react with the alkaline material in ash [66].

As methanogens adapt poorly to pH fluctuations, either a high or a low pH had the same effects on microbes in terms of functional inhibition. Table 4 shows that an increase in OLR of 4 kg VS/m^3^·d was followed by an alkaline pH at about 8.16 ± 0.28, which might hinder methanogen progress with reference to Song et al., (2020), who reported that a pH range of 6.8–7.5 was required for healthy microbial growth [58]. Certainly, pH variation definitely impacted methanogenesis progress. However, at an OLR of 4.5 kg VS/m^3^·d, the identified neutral range of pH was not a powerful indicator to determine whether or not a healthy balance between microbial populations and a steady process was achieved. Indeed, at an OLR of 4.5 kg VS/m^3^·d, a decline in terms of SBY of around 46% was noticed. This was caused by an increase of 30% in the VFAs concentration, implying an overcharge of R3 (with a VFA:TAC ratio exceeding 1) and a drop of methane content by 50%. Ultimately, the SBY and SMY recorded during the OLR variations clearly indicated a synergistic relationship between FW and UBc, which is in agreement with several previous works. However higher OLRs, as well as better SMY, were achievable [36,61,65]. This divergence might be due to the experimental conditions relating to biochar production, such as the type of feedstock used, temperature, pressure, etc., as well as the dose of biochar that was added to the anaerobic digesters [66]. Eventually, either a combination of agricultural waste or industrial waste improved, under different operational conditions, SMYs during the entire AD run. This confirmed that the addition of co-substrates not only sustained a performant process in terms of SMY, but also boosted methanogen activity, and then in-situ biogas upgrading [67].

#### 4.3.3. Co-Substrate Addition Effects on Digestate Characteristics

Once the anaerobic treatment had been accomplished, the generated digestates were collected for characterization. However, a comparison between the digestate properties was undertaken during the work to identify whether or not the gathered AD residues met the required physio-chemical criteria, allowing them to be safely spread on land, or if further AD effluent post-treatments were needed. Therefore, several parameters were identified in order to determine suitable indicators, and to assess the potential agricultural use of the digestates. Accordingly, the various physio-chemical characteristics of D1, D2, and D3 were examined (Table 6).

First, pH ranged around neutral values for D1 and D2, which was beneficial for a direct spread onto land [67], while an alkaline pH of around 8.53 marked D3. In fact, the alkaline pH of the R3 digester was expected, due to the alkaline nature of the added UBc, as well as its relatively high ash content. Similar findings were reported by Shen et al., (2016) [68], revealing that the biochar derived from woody substrates had a significant impact on the pH tendencies of the AD by-products. However, such a relatively high pH implied ammonium emissions when it was directly spread out onto land [69], which imposed post-treatment in the case of D3. Moreover, the alkaline nature of UBc impacted also the conductivity (EC) of D3, which was 91% and 124% higher than R1 and R2, respectively, and reached 11.73 mS/cm. However, that does not deny the fact that R1 and R2 were, in addition, characterized by a relatively high conductivity EC (>4 mS/cm), at about 5.23 and 6.12 mS/cm, respectively. As this latter could prove toxic for plants, all the generated digestates were restricted from being directly used as biofertilizer [70]. This was because one of the steering factors, MC, was firstly identified, and a relatively high-water content of around 97% was found to mark all the digesters. In practice, high levels of moisture cause certain concerns such as odor, cost-intensive transport, and the need for hard storage facilities. The generated AD residues were characterized by almost significant water content, from which rose the idea of exploiting it as an unconventional moisturizing agent (MA), feeding in-vessel composters. However, above all, various criteria were fixed, including macro- and micro-nutrient availability, as well as the heavy metals content of each digestate, to decide later whether or not AD effluents were efficacious as unconventional MAs and could act as an efficient inoculum for FW aerobic treatment. These are aspects that will be considered in future research.

## 5. Conclusions and Perspectives

To manage organic residues efficiently and unlock the full sustainability of anaerobic digestion potential, this research aimed to develop a closed cycle “biowaste to bioenergy” process, treating mainly FW and exploiting various types of organic residues generated from different sectors of activity. A mixture of carbonaceous substrates (WS) and a rich-nitrogenous one (CM) was chosen to represent the agricultural sector, while unmarketable biochar (UBc), which also had similar features, took the form of industrial residue. Operating under variable OLRs, R2 and R3 illustrated different SBY and SMY profiles. In fact, by the end of the start-up conditions, both R2 and R3 demonstrated higher biogas production, reaching 403 L_N_/kg VS_in_ and 369.7 L_N_/kg VS_in_, qualified by 66.33% and 68.77% of CH_4_, respectively. Thus, an enhanced process performance was identified compared to mono-digested FW. Among variable OLRs, a relatively low OLR that did not exceed 3 kg VS/m^3^·d was considered as the most appropriate range for anaerobic reactors fed with agricultural residues, while higher OLRs were convenient for anaerobic reactors amended with unmarketable biochar. Indeed, higher biogas and methane yields were noted for an organic charge of 4 kg VS/m^3^·d, to be around 507.0 L_N_/kg VS_in_ and 342.1 L_N_/kg VS_in_, respectively. However, the increase in the OLR to 4.5 kg VS/m^3^·d was followed by an increase in the VFA:TAC ratio to 1.21 ± 0.26, and in turn, a decrease in both SBY and SMY marked R3. Hence, the results demonstrated that the selected co-substrates met the targeted requirements in terms of FW anaerobic co-digestion performance and AD-effluent (biogas and digestate) upgrading, as well as organic waste management sustainability. However, the outcomes of the current research are additionally aimed at providing decision makers with sufficient technical details to design biogas and composting plants. As the selected co-substrates (WS + CM) and UBc boosted the AcoD processing of FW, further criteria need to be considered before the implementation of biogas and/or composting plants:

- A roadmap is definitely required to highlight the accessibility of the selected co-substrates;
- An in-depth review of various indicators such as socio-economic and environmental aspects is obligatory;
- Appropriate biogas and/or composting plant design (in terms of capacity, OLR, HRT, cost, etc.) is mandatory to optimize energy and compost use.

## Figures and Tables

**Table 1:** Experimental setup design.

Phase	Feeding of Digesters			Organic Loading Rate (kg VS/m3·d)	Period (Days)
	R1: FW	R2: FW:CM:WS	R3: FW:UBc		
Phase I	+	+	+	2	1–61
Phase II	+	+	+	2.5	62–92
	−	+	+	3	93–123
	−	+	+	3.5	124–154
	−	+	+	4	155–185
	−	−	+	4.5	186–216

(+): Feeding; (−): Feeding stopped.

**Table 2:** Physical and chemical parameter measurement of biowastes chosen for biological treatment.

Parameters	Units	Method of Analysis	Reference
pH	-	(1:10 *w*/*v* sample: water extract)	ISO 10390 (1994)
Moisture content (MC)	% of FM ^1^	Using electronic oven by drying at 105 °C for 24 h	NF ISO 11465 (1994)
Total solids (TS)	% of FM ^1^		
Total carbon (TC)	% of FM ^1^	TOC (%) = ((100 − Ash%) ÷ 1/8)	[44]
Total nitrogen (TN)	% of FM ^1^	Titrimetric methods	NF ISO 11265 (1995)
Phosphorus (P)	% of TS ^2^	Atomic absorption spectrometric methods	ISO 11885 (2007)
Potassium (K)	% of TS ^2^		
Magnesium (Mg)	% of TS ^2^	Spectrometer, Thermo-Elemental ICP MS-X Series	ISO 11885 (2007)
Lead (Pb)	mg/kg TS	Spectrometer, Thermo-Elemental ICP MS-X Series	ISO 11885 (2007)
Copper (Cu)	mg/kg TS	Spectrometer, Thermo-Elemental ICP MS-X Series	ISO 11885 (2007)
Zinc (Zn)	mg/kg TS	Spectrometer, Thermo-Elemental ICP MS-X Series	ISO 11885 (2007)
Nickel (Ni)	mg/kg TS	Spectrometer, Thermo-Elemental ICP MS-X Series	ISO 11885 (2007)
Cadmium (Cd)	mg/kg TS	Spectrometer, Thermo-Elemental ICP MS-X Series	ISO 11885 (2007)
Arsenic (As)	mg/kg TS	Spectrometer, Thermo-Elemental ICP MS-X Series	ISO 11885 (2007)

^1^ FM: fresh matter; ^2^ TS: total solids.

**Table 3:** Physio-chemical characteristics of the raw materials.

Parameters	Units	FW	UBc	CM	WS
pH	-	4.22	10.21	8.01	-
Conductivity (EC)	(mS/cm)	5.71	12.73	-	-
Total solids	% of ^1^ FM	26.00	97.60	7.10	91.10
Volatile solids	% of ^2^ TS	94.60	83.00	80.30	86.80
Carbon (C)	% of FM	20.52	52.47	42.61	47.63
Nitrogen (N)	% of FM	1.20	-	1.70	0.61
C:N ratio	-	17.10	-	25.64	78.08
Phosphorous (P)	% of TS	0.48	0.27	0.60	0.06
Potassium (K)	% of TS	0.91	5.21	2.95	1.74
Magnesium (Mg)	% of TS	0.09	2.92	2.82	0.25
Calcium (Ca)	% of TS	0.06	0.57	0.61	0.07
Lead (Pb)	mg/kg TS	0.91	1.10	0.85	0.21
Copper (Cu)	mg/kg TS	6.82	9.86	18.20	1.78
Zinc (Zn)	mg/kg TS	16.33	8.10	131.00	16.6
Nickel (Ni)	mg/kg TS	0.95	9.34	6.91	5.78
Cadmium (Cd)	mg/kg TS	0.07	<0.01	0.19	0.08
Chrome (Cr)	mg/kg TS	2.31	67.80	-	10.50
Arsenic (As)	mg/kg TS	0.57	0.41	0.28	0.07
Mercury (Hg)	mg/kg TS	<0.01	0.03	<0.01	<0.01

^1^ FM: fresh matter; ^2^ TS: total solids.

**Table 4:** Anaerobic process performance indicators during OLR variation (mean ± standard deviation (SD)).

Feedstock	OLR (kg VS/m^3^·d)	VFA (mg/L)	TAC (mg CaCO_3_/L)	VFA: TAC
R1: FW_100_	2.0	^a^ 1562.06 ± 212.01	^a^ 9466.06 ± 2769.74	^a^ 0.17 ± 0.04
	2.5	^b^ 2461.63 ± 456.42	^b^ 4212.04 ± 618.71	^b^ 0.60 ± 0.18
	3.0	^b^ 3415.25 ± 529.02	^b^ 2167.75 ± 968.42	^b^ 1.89 ± 0.98
	3.5	-	-	-
	4.0	-	-	-
	4.5	-	-	-
R2: FW_60_CM_20_WS_20_	2.0	^a^ 1907.06 ± 264.84	^a^ 9950.13 ± 3322.24	^a^ 0.21 ± 0.07
	2.5	^b^ 2151.88 ± 250.88	^b^ 5320.75 ± 769.86	^b^ 0.38 ± 0.03
	3.0	^b^ 2369.20 ± 118.72	^b^ 4995.45 ± 506.66	^b^ 0.47 ± 0.01
	3.5	^b^ 2761.30 ± 217.08	^b^ 4112.56 ± 277.58	^b^ 0.67 ± 0.04
	4.0	^b^ 2999.00 ± 320.04	^b^ 3575.63 ± 854.06	^b^ 0.89 ± 0.28
	4.5	-	-	-
R3: FW_90_UBc_10_	2.0	^a^ 1840.38 ± 230.67	^a^ 10207.44 ± 2531.58	^a^ 0.19 ± 0.03
	2.5	^b^ 2050.50 ± 258.47	^b^ 7065.13 ± 228.16	^b^ 0.29 ± 0.04
	3.0	^b^ 2035.00 ± 55.92	^b^ 7191.50 ± 451.52	^b^ 0.28 ± 0.01
	3.5	^b^ 2028.75 ± 257.46	^b^ 6068.00 ± 348.09	^b^ 0.34 ± 0.04
	4.0	^b^ 3115.00 ± 932.08	^b^ 5842.50 ± 398.62	^b^ 0.53 ± 0.13
	4.5	^b^ 4051.25 ± 276.52	^b^ 3351.00 ± 437.15	^b^ 1.21 ± 0.26

^a^ Values are means of data recorded during the 8 weeks of the experiment ± SD; ^b^ Values are means of data recorded during the 4 weeks of the experiment ± SD; (-) No measurement of process performance indicators.

**Table 5:** In-situ biogas up vs. pH fluctuations (mean ± standard deviation (SD)).

Feedstock	OLR (kg VS/m^3^·d)	pH	SMY (L_N_/kg VS_in_)	% CH_4_
R1: FW_100_	2.0	^a^ 7.31 ± 0.61	^a^ 150.63 ± 31.39	^a^ 57.02 ± 5.54
	2.5	^b^ 7.05 ± 0.75	^b^ 135.56 ± 25.31	^b^ 58.15 ± 5.41
	3.0	^b^ 6.22 ± 0.35	^b^ 57.42 ± 24.51	^b^ 34.66 ± 9.64
	3.5	-	^b^ 23.54 ± 4.21	-
	4.0	-	-	-
	4.5	-	-	-
R2: FW_60_ CM_20_WS_20_	2.0	^a^ 7.37 ± 0.41	^a^ 233.03 ± 15.61	^a^ 61.99 ± 2.18
	2.5	^b^ 7.28 ± 0.49	^b^ 294.92 ± 24.96	^b^ 65.56 ± 1.64
	3.0	^b^ 7.04 ± 0.36	^b^ 282.22 ± 74.09	^b^ 64.67 ± 4.39
	3.5	^b^ 6.70 ± 0.12	^b^ 151.36 ± 21.93	^b^ 55.09 ± 2.27
	4.0	^b^ 6.05 ± 0.17	^b^ 47.95 ± 20.93	^b^ 29.27 ± 5.61
	4.5	-	^b^ 20.60 ± 3.85	-
R3: FW_90_UBc_10_	2.0	^a^ 7.91 ± 0.20	^a^ 208.28 ± 34.73	^a^ 66.49 ± 2.23
	2.5	^b^ 7.86 ± 0.20	^b^ 279.93 ± 16.92	^b^ 71.08 ± 1.51
	3.0	^b^ 7.90 ± 0.29	^b^ 305.04 ± 12.02	^b^ 70.63 ± 4.04
	3.5	^b^ 7.49 ± 0.39	^b^ 352.02 ± 10.97	^b^ 72.37 ± 2.28
	4.0	^b^ 8.16 ± 0.28	^b^ 289.71 ± 41.51	^b^ 64.49 ± 2.64
	4.5	^b^ 7.14 ± 0.43	^b^ 137.52 ± 34.57	^b^ 48.42 ± 3.77

^a^ Values are the mean of data recorded during 8 weeks of the experiment ± SD; ^b^ Values are the mean of data recorded during the 4 weeks of experiment ± SD; (-) No measurement of process performance indicators.

**Table 6:** Physio-chemical properties of AD effluents.

Parameters	Units	D1	D2	D3
pH	-	7.67	7.79	8.53
Conductivity (EC)	mS/cm	5.23	6.12	11.72
Moisture content (MC)	% of ^1^ FM	97.50	97.60	97.30
Crude ash	% of ^2^ TS	32.30	36.40	44.10
Carbon (C)	% of FM	40.10	35.20	47.60
Nitrogen (N)	% of FM	4.70	3.70	3.20
C:N ratio	-	8.53	9.51	14.88
Phosphorous (P)	% of TS	3.87	4.17	4.91
Potassium (K)	% of TS	5.21	5.04	11.86

^1^ FM: fresh matter; ^2^ TS: total solids.

## Data Availability

Data available in a publicly accessible repository.
